# Hepatitis C infection and chronic kidney disease among Hispanics/Latinos

**DOI:** 10.1097/MD.0000000000028089

**Published:** 2021-12-10

**Authors:** Eugenia Wong, Ana C. Ricardo, Sylvia E. Rosas, James P. Lash, Nora Franceschini

**Affiliations:** a Department of Epidemiology, Gillings School of Global Public Health, University of North Carolina at Chapel Hill, Chapel Hill, NC; b Department of Medicine, University of Illinois at Chicago, Chicago, IL; c Joslin Diabetes Center and Beth Israel Deaconess Medical Center, Harvard Medical School, Boston, MA.

**Keywords:** chronic kidney disease, hepatitis C, minority health, Viral Infections

## Abstract

Viral infections, including hepatitis C, can cause secondary glomerular nephropathies. Studies suggest that hepatitis C virus infection (HCV+) is a risk factor for chronic kidney disease (CKD) but evidence of this relationship is lacking among Hispanics/Latinos. We examined the association between HCV+ and incident CKD in a prospective cohort of Hispanics/Latinos enrolled in the Hispanic Community Health Study/Study of Latinos. HCV+ was defined by detectable HCV antibodies with additional confirmation through HCV RNA or recombinant immunoblot assay testing. Incident CKD was defined by an estimated glomerular filtration rate (eGFR) <60 mL/min/1.73 m^2^ or sex-specific threshold for albuminuria measured during follow-up. We used Poisson regression to estimate incidence rate ratios (IRR) of CKD and changes in eGFR- or albuminuria-based risk stages, separately. We used linear regression to estimate associations with continuous, annualized changes in eGFR and albuminuria.

Over a follow-up period of 5.9 years, 712 incident CKD events occurred among 10,430 participants. After adjustment for demographic characteristics and comorbidities, HCV+ was not associated with incident CKD, defined by eGFR and albuminuria thresholds (IRR 1.29, 95% Confidence Interval 0.61, 2.73). HCV+ was significantly associated with higher eGFR risk stages (IRR 2.39, 95% CI 1.47, 3.61) with most participants transitioning from stage G1 to G2. HCV+ was associated with a continuous, annualized eGFR decline of −0.69 mL/min/m^2^/year (95% CI −1.23, −0.16). This large, cohort study did not find evidence of a strong association between HCV+ and new-onset CKD among Hispanics/Latinos. HCV infection may not be associated with risk of CKD among Hispanics/Latinos, although treatment with direct-acting antivirals is recommended for all HCV+ individuals, including those with established CKD or end-stage kidney disease.

## 1 Introduction

Recent estimates indicate that 2.4 million adults in the United States are currently infected with hepatitis C virus (HCV+).^[1]^ While HCV is most commonly recognized as a risk factor for liver disease, chronic HCV infection has been associated with secondary glomerular nephropathies, including membranoproliferative glomerulonephritis and cryoglobulinemia,^[2,3]^ and chronic kidney disease (CKD). Although the biological mechanisms involved are not fully understood, HCV may drive the development of kidney disease directly, by binding to renal cells,^[3]^ or indirectly, by galvanizing a systemic immune response, leading to an accumulation of immune complexes in the kidneys that eventually disrupts normal function.^[4]^

Previous works have explored the impact of HCV on risk of CKD, but studies have often used different case definitions of both HCV+ and reduced kidney function.^[5–8]^ For example, many studies have defined HCV+ solely based on antibody testing, which cannot distinguish between resolved versus active, chronic infection.^[9]^ In one retrospective study of veterans, those testing positive for HCV antibodies and viremia had a 10% greater incidence of estimated glomerular filtration rate (eGFR) <60 mL/min/1.73 m^2^ and a 62% greater risk of end-stage kidney disease (ESKD) when compared to those without HCV antibodies.^[10]^ However, this result was no longer significant when investigators defined HCV using only antibody results, without regard to viremia.^[6]^ Importantly, the present study aims to address these gaps in the literature by using more stringent conditions to define both HCV+ and CKD to better understand the potential association between chronic HCV infection and incident CKD. HCV+ will be defined using RNA testing in addition to antibody results, and cases of CKD will be identified using both eGFR and albuminuria.

Additionally, this study aims to address the relative lack of studies investigating the link between infectious diseases and kidney disease in minority populations, including Hispanic/Latinos. Approximately 4% of Hispanics/Latinos have chronic HCV, and in comparison to non-Hispanic whites, they also have a greater risk of developing cirrhosis and are 40% more likely to die from HCV-related causes.^[11–13]^ These findings suggest that Hispanics/Latinos may face heightened risks of HCV progression and complications, including kidney disease. However, previous studies of the association between HCV and CKD have included few Hispanic/Latino participants.^[14–16]^ Therefore, our primary objective was to describe the relationship between HCV+ and the incident CKD in the Hispanic Community Health Study/Study of Latinos (HCHS/SOL), the largest prospective study ever conducted in individuals of Hispanic or Latino heritage living in the United States. Our secondary objectives were to estimate risks of decreased eGFR and increased albumin-to-creatinine ratio (ACR), separately, by examining both categorical (risk stage-based) and continuous changes in kidney function associated with HCV infection.^[17]^

## 2 Methods

### 2.1 Study population

The HCHS/SOL study is a multi-center, prospective cohort of Hispanic/Latino adults from across the United States. Details of the study design have been published elsewhere.^[18]^ Briefly, 16,415 individuals of 18 to 75 years of age were enrolled from 2008 to 2011 using stratified, two-stage, area probability sampling of census block groups and households in four centers: Chicago, IL; Miami, FL; Bronx, NY; San Diego, CA. Sampling weights were generated to reflect probabilities of selection into the study. During visit 1 (2008–2011), a clinical exam was performed, biological samples were collected, and data on a variety of demographics and risk factors was gathered by questionnaire. Approximately 6 years after completing visit 1, study participants were invited to complete a second clinical examination (visit 2; 2015–2017), during which these measures were collected again.^[19]^ The retention rate of the study at visit 2 was high (81%), although some participants (n = 1,224) were administratively excluded from participation because they had relocated outside of the United States or moved more than 100 miles from the nearest study field center since visit 1.^[19]^ In a prior investigation of retention in the HCHS/SOL study, 38% of visit 2 participants reported challenges to study participation, including family and work obligations and lack of residential stability; these factors likely contributed to loss to follow-up among those who did not complete visit 2.

**Figure 1:**
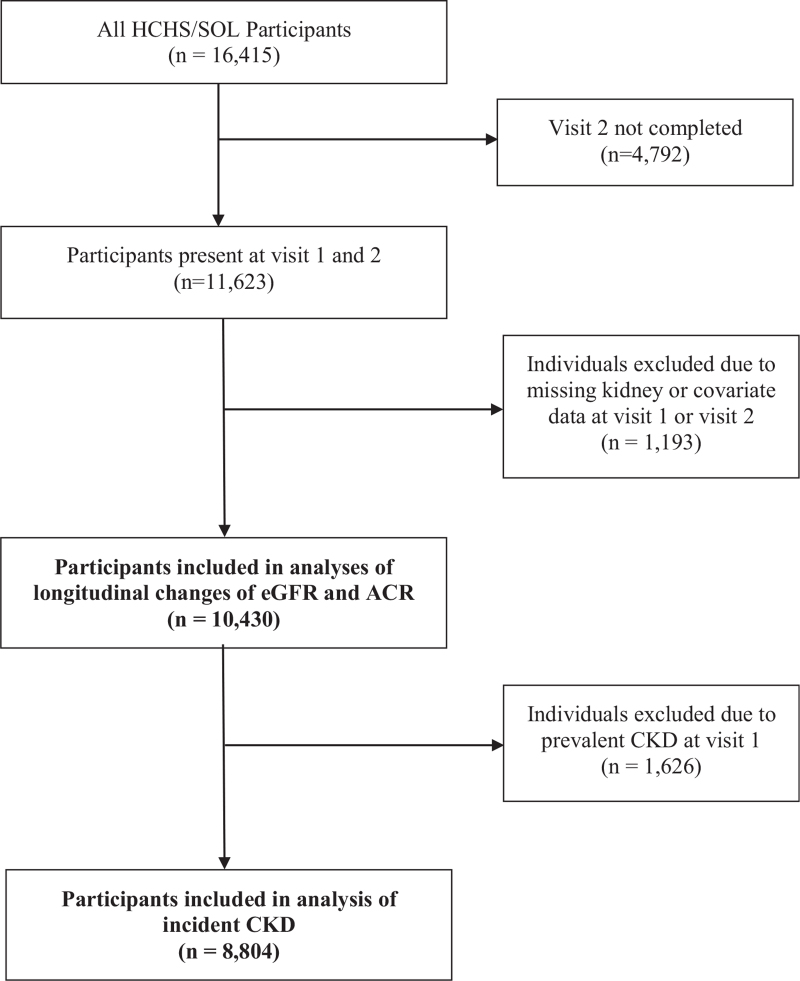
Study population. ACR = albumin-to-creatinine ratio, CKD = chronic kidney disease, eGFR = estimated glomerular filtration rate, HCHS/SOL = Hispanic Community Health Study/Study of Latinos.

In the present analysis, 11,623 participants who completed visit 1 and visit 2 were eligible for inclusion (Fig. 1). Participants were excluded if they had missing HCV data at visit 1 (n = 5), or missing measurements of serum creatinine (n = 175) or ACR (n = 703) at either visit. Further exclusions were made if participants were missing covariate data regarding Hispanic background, hypertension, diabetes, cigarette and alcohol use, or education (n = 310, mostly due to missing blood pressure data). The final analytic population consisted of 10,430 participants. All those with prevalent CKD at visit 1 were excluded from the main analysis of incident CKD (n = 1,626), leaving 8,804 participants in the investigation of this outcome. Longitudinal changes in eGFR and ACR were studied in all 10,430 participants. This study was conducted in accordance with the Declaration of Helsinki and the HCHS/SOL protocol was approved by the institutional review board of each field center in Chicago, IL (Northwestern University), Miami, FL (University of Miami), Bronx, NY (Albert Einstein College of Medicine) and San Diego, CA (San Diego State University). All participants provided written, informed consent.

### 2.2 HCV testing at visit 1

HCV antibody was first measured in serum using an indirect, two wash immunoassay on the ADVIA Centaur System (Siemens Healthcare Diagnostics, Deerfield, IL 60015–0778). In immunoassays that tested positive, HCV was quantitated in serum using real-time polymerase chain reaction (PCR) on the ABI 7000 Analyzer, Applied BioSystems, Foster City, CA 94404) to confirm the presence of HCV RNA. Among samples in which the HCV antibody signal to cut-off ratio was >1.0 and <11.0, and HCV by polymerase chain reaction was <25, confirmatory recombinant immunoblot assay tests were performed at ARUP Laboratories, Salt Lake City, UT 84108 prior to May 10, 2011, after which they were performed at Mayo Medical Laboratory, Rochester, MN 55901. Only participants who tested positive for HCV antibodies and were confirmed with HCV RNA/recombinant immunoblot assay testing were considered HCV+.

### 2.3 Kidney measures at visits 1 and 2

Urinary albumin was measured using an immunoturbidometric method on the ProSpec nephelometric analyzer (Dade Behring GMBH. Marburg, Germany D-35041). Serum and urinary creatinine were measured on a Roche Modular P Chemistry Analyzer (Roche Diagnostics Corporation) using a creatinase enzymatic method (Roche Diagnostics, Indianapolis, IN 46250). eGFR was calculated using the 2009 CKD Epidemiology Collaboration creatinine equation.^[20]^ Serum cystatin C was measured using a turbidimetric method on the Roche Modular P Chemistry Analyzer (Gentian AS, Moss, Norway) and eGFRcys was calculated based on the 2012 CKD-EPI cystatin C equation.^[21,22]^ ACR was estimated from measures of urinary albumin and creatinine.

### 2.4 Assessment of covariates at visit 1

Interviews and clinical examinations were performed to collect demographic data, such as Hispanic background, and information regarding health behaviours and medical history. Hypertension was defined as having a systolic blood pressure ≥130 mm Hg and diastolic blood pressure ≥80 mm Hg^[23]^ or use of any anti-hypertensive medication. Diabetes mellitus was defined by having a fasting blood glucose of ≥126 mg/dL or a 2-h post-load glucose level of ≥200 mg/dL, or a hemoglobin A1c level of ≥6.5%, per American Diabetes Association guidelines,^[24]^ or by participants’ self-reported use of anti-diabetic medication. Body mass index was calculated by dividing study measurements of weight (kg) by squared height (m^2^). Participants were categorized as either former, never, or current cigarette smokers and low, moderate, or high users of alcohol, based on questionnaire responses. The number of HCV+ individuals in some Hispanic background groups was low. To address this issue, Hispanic background groups were collapsed into a dichotomous Caribbean category (yes if Dominican, Cuban, or Puerto Rican; no if Mexicans, Central American, South American, more than one heritage, other).

### 2.5 Outcomes

The primary outcome of incident CKD at visit 2 was defined as having eGFR <60 mL/min/m^2^ or sex-specific cut-offs of albuminuria: ACR > 17 mg/g in men, ACR > 25 mg/g in women, among participants without prevalent CKD.^[25]^

The second outcome was changes in eGFR. To examine categorical decreases in eGFR as per KDIGO guidelines, eGFR was divided into five risk stages: ≥90 (G1), 60 to 89 (G2), 45 to 59 (G3a), 30 to 44 (G3b), <30 (G4+) mL/min/1.73 m^2^. A participant was categorized as having worsened eGFR if at visit 2, the participant had moved into a higher eGFR risk category than at visit 1—for example, G2 at visit 1 to G4 at visit 2. Continuous, annualized changes in eGFR (Δ mL/min/m^2^ per year) were also assessed. As a sensitivity analysis, analogous changes in cystatin-based eGFR were investigated; those missing measures of serum cystatin C (n = 73) were excluded.

The third outcome was changes in ACR. Based on Kidney Disease Improving Global Outcomes (KDIGO) classifications,^[17]^ participants were divided into 3 risk stages of albuminuria: <30 (A1), 30 to 300 (A2), >300 (A3) mg/g. Individuals were defined as having increased ACR, if they moved to a higher ACR stage from visit 1 to visit 2.

### 2.6 Statistical analysis

Crude and adjusted incidence rate ratios (IRR) of CKD, and decreased eGFR and increased ACR stages associated with HCV+ were estimated using Poisson regression. Models were adjusted for demographic factors (sex, age, Caribbean origin, educational attainment, field center) and further adjusted for health behaviours and comorbidities (smoking, alcohol use, body mass index, hypertension, diabetes). To explore potential effect measure modification, the effects of HCV+ on incident CKD, stages of eGFR and ACR were also estimated across strata of sex, age, Caribbean origin, diabetes, and hypertension. The interaction between HCV+ and each of these variables was also tested using likelihood ratio tests at a significance level of α = 0.05. The association between HCV+ and annualized changes in eGFR and ACR were examined using multiple linear regression, controlling for participants’ baseline (visit 1) measures in addition to the above covariates. All analyses were performed using Stata, version 15 (StataCorp).

## 3 Results

### 3.1 Study population characteristics at visit 1

In this analysis, 10,430 participants had a median follow-up of 5.9 years between visits 1 and 2 (Fig. 1). Among study participants, 112 (1.1%) individuals were HCV+ at visit 1. HCV+ participants were much more likely to be female, of Caribbean origin, and current smokers in comparison to those that were HCV− (Table 1). HCV+ individuals also tended to be less educated and low-level users of alcohol. The prevalence of diabetes and hypertension were both greater in HCV+ individuals (by 9% and 12%, respectively). HCV+ participants had a higher median ACR (8.7 mg/g, IQR: 15.5) compared to HCV− individuals (6.67 mg/g, IQR: 7.5) (Table 1) and prevalent CKD was more common among HCV+ than HCV− individuals. However, ACR was highly variable in both groups.

**Table 1:** Study population characteristics by hepatitis C status at visit 1.

	Total	HCV+	HCV−
N	10,430	112	10,318
Age, years (SD)	47.3 (13.3)	53.5 (7.9)	47.2 (13.3)
Female	3,883 (37.2%)	63 (56.3%)	3,820 (37.0%)
Caribbean^∗^	6,477 (61.1%)	69 (61.6%)	3,984 (38.6%)
Education			
<High school	3,935 (37.7%)	47 (42.0%)	3,888 (37.7%)
High school	2,620 (25.1%)	39 (34.8%)	2,581 (25.0%)
>High school	3,875 (37.2%)	26 (23.2%)	3,849 (37.3%)
Smoking			
Never	6,439 (61.7%)	30 (26.8%)	6,409 (62.1%)
Former	2,123 (20.4%)	29 (25.9%)	2,094 (20.3%)
Current	1,868 (17.9%)	53 (47.3%)	1,815 (17.6%)
Alcohol use			
Low	5,600 (53.7%)	66 (58.9%)	5,534 (53.6%)
Moderate	4,358 (41.8%)	38 (33.9%)	4,320 (41.9%)
High	472 (4.5%)	8 (7.1%)	464 (4.5%)
BMI, kg/m^2^ (SD)	30.0 (6.0)	28.7 (5.9)	30.0 (6.0)
Diabetes	2,185 (20.9%)	33 (29.5%)	2,152 (20.9%)
Hypertension	3,204 (30.7%)	48 (42.9%)	3,156 (30.6%)
HDL, mg/dL (SD)	49.2 (12.9)	47.7 (13.4)	49.2 (12.9)
LDL, mg/dL (SD)	123.8 (36.7)	97.5 (31.1)	124.1 (36.6)
Alanine aminotransferase, U/L (SD)	27.4 (21.9)	65.0 (65.8)	27.0 (20.6)
Aspartate aminotransferase, U/L (SD)	24.4 (15.3)	60.6 (58.8)	24.0 (13.6)
Visit 1 eGFR, mL/min/m^2^			
≥90 (G1)	6,910 (66.3%)	66 (58.9%)	6,844 (66.3%)
60–89 (G2)	3,195 (30.6%)	44 (39.5%)	3,151 (30.5%)
45–59 (G3a)	248 (2.4%)	2 (1.8%)	246 (2.4%)
30–44 (G3b)	56 (0.5%)	0 (0%)	56 (0.5%)
<30 (G4+)	21 (0.2%)	0 (0%)	21 (0.2%)
Visit 1 ACR, mg/g			
<30 (A1)	9,353 (89.7%)	89 (79.5%)	9,264 (89.8%)
30–300 (A2)	920 (8.8%)	15 (13.4%)	905 (8.8%)
>300 (A3)	157 (1.5%)	8 (7.1%)	149 (1.4%)
Prevalent CKD at Visit 1	1,626 (15.6%)	31 (27.7%)	1,595 (15.5%)
Incident CKD at Visit 2	712 (6.8%)	13 (11.6%)	699 (6.5%)

### 3.2 Incidence of CKD

Among 8,804 participants without CKD at baseline, 712 (6.8%) participants developed incident CKD at visit 2, with 13 events occurring in the HCV+ group and 699 events in the HCV− group (Table 1). In crude analyses, HCV+ was associated with a 75% increase in the incidence of CKD: (IRR 1.75, 95% CI 0.89, 3.44) (Table 2). The IRR was attenuated (IRR 1.29, 95% CI 0.61, 2.73) after adjustment for demographics (sex, age, Caribbean origin, educational attainment, field center), comorbidities and health behaviors (smoking, alcohol use, body mass index, hypertension, diabetes) previously found to be risk factors for CKD in this cohort.^[26]^

**Table 2:** Effects of hepatitis C on incidence of impaired kidney function.

	Events		Incidence Rate Ratios		
	HCV–	HCV+	Model 1	Model 2	Model 3
CKD^∗^	699	13	1.75 (0.89, 3.44)	1.21 (0.61, 2.40)	1.29 (0.61, 2.73)
+eGFR stage^∗∗^	1016	28	3.87 (2.35, 6.38)	2.40 (1.55, 3.74)	2.39 (1.47, 3.61)
+ACR stage^∗∗^	648	11	1.34 (0.65, 2.79)	0.97 (0.48, 2.01)	0.91 (0.42, 1.96)

### 3.3 Changes in KDIGO eGFR stage

Overall, 2,277 (21.8%) individuals experienced a change in eGFR stage, with 1,044 (10.0%) participants moving to a stage of worsened eGFR (eTable 1). Increases in eGFR stage occurred most commonly among participants who had normal to high eGFR at visit 1 (≥90 mL/min/m^2^, stage G1, 7.0%) and then experienced moderately decreased eGFR at visit 2 (60–89 mL/min/m^2^, stage G2). Very few individuals experienced a worsening of eGFR beyond a single stage increase. The proportion of individuals who experienced worsened eGFR was greater among HCV+ participants: 25.0% of HCV+ versus 9.8% of HCV− individuals had a worsened eGFR stage by visit 2. The crude incidence of worsened eGFR stage was nearly 4 times higher among HCV+ than HCV− participants (IRR 3.87, 95% CI 2.35, 6.38). Even in fully adjusted models, incidence of worsened eGFR stage was more than double in the HCV+ versus HCV− group (IRR 2.39, 95% CI 1.47, 3.61) (Table 2).

### 3.4 Yearly changes in eGFR

Linear models of annualized changes in eGFR showed results similar to those reflected by stage-based classifications of decline. In both HCV+ and HCV− participants, mean eGFR decreased between visits 1 and 2. However, in comparison to HCV− participants, HCV+ was associated with an adjusted decrease of −0.69 mL/min/m^2^ per year (95% CI, −1.23, −0.16) (Table 3).

**Table 3:** Effects of hepatitis C infection on annual changes in kidney function.

	Mean change		Change associated with HCV+		
	HCV–	HCV+	Model 1	Model 2	Model 3
Δ eGFR^∗^(mL/min/m^2^)/year	−0.06	−1.16	−1.35 (−1.94, −0.75)	−0.68 (−1.20, −0.15)	−0.69 (−1.23, −0.16)
ΔACR^∗∗^(mg/g)/year	2.98	−5.38	−1.11 (−5.79, 3.58)	−3.49 (−8.50, 1.52)	−3.61 (−8.71, 1.49)

### 3.5 Sensitivity analyses using cystatin C-based eGFR (eGFRcys)

The relationship between HCV+ and CKD was also assessed using eGFRcys, based on the 2012 CKD-EPI cystatin C equation.^[21,22]^ Slightly more HCV+ individuals were classified as having CKD by the cystatin C equation (eTable 2). In crude analyses, HCV+ was significantly associated with cystatin C-based incident CKD (IRR 2.23, 95% CI 1.22, 4.11) but this association was attenuated after adjustment (IRR 1.58, 95% CI 0.81, 3.11).

For analyses of KDIGO stages, the overall impact of HCV+ was similar to eGFRcr estimates. The crude IRR of increased eGFRcys stage associated with HCV+ was 2.46 (95% CI 1.67, 3.63) and remained significant after adjustment (IRR 1.50, 95% CI 1.06, 2.12) (eTable 2). HCV+ was associated with decrease in eGFRcys of −0.82 mL/min/m^2^ per year compared to HCV− (95% CI −1.45, −0.18) in fully adjusted models, demonstrating a stronger relationship when eGFR was estimated using cystatin C versus creatinine.

### 3.6 Changes in KDIGO ACR stage

By visit 2, 1,150 (11.0%) individuals had changed ACR stages with 659 (6.3%) who had progressed to a higher ACR stage (eTable 3). Most participants moved from stage A1 at visit 1 to stage A2 at visit 2. Increases in albuminuria stage occurred in 8.9% of HCV+ versus 6.3% of HCV− participants. In comparison to HCV− individuals, the crude IRR of categorically increased ACR was 1.34 (95% CI 0.65, 2.79). This association was no longer statistically significant, after adjusting for demographics and comorbidities (IRR 0.91 95% CI 0.42, 1.96) (Table 2). However, ACR was highly variable in our study population and estimates were imprecise (Table 3).

### 3.7 Impact of HCV+ on eGFR in population subgroups

**Figure 2:**
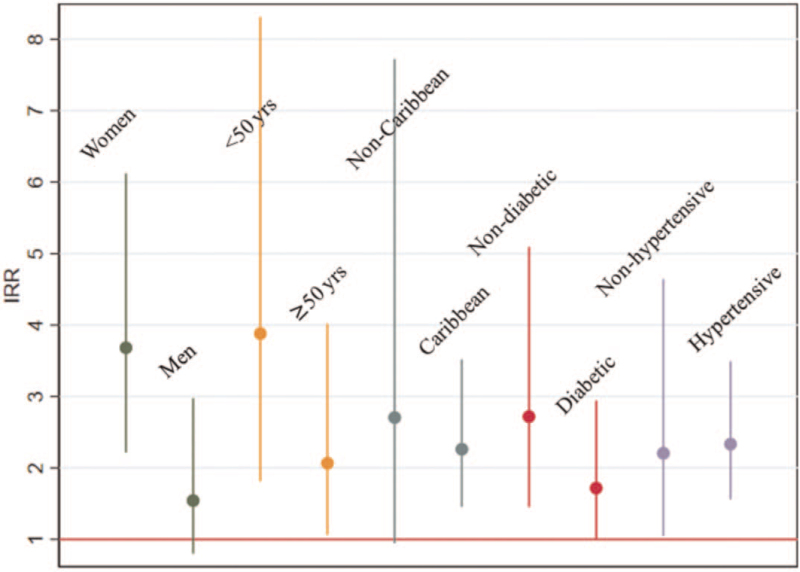
Incidence of decreased eGFR associated with HCV+ in population subgroups. Incidence rate ratios of decreased eGFR (a categorical change in eGFR stage) were generated using Poisson regression, stratified by dichotomous subgroups of sex (dark green), age (orange), Caribbean background (light green), diabetes mellitus (red), and hypertension (purple). In formal tests of interaction, sex was the only factor by which the impact of HCV+ varied significantly in fully adjusted analyses (*P* for interaction = .01).

Of note, HCV+ was associated with decreased KDIGO stage-based eGFR in women (IRR 3.68, 95% CI 2.21, 6.12) but not men (IRR 1.54, 95% CI 0.80, 2.97) (Fig. 2). HCV+ was associated with decreased eGFR in both those under the age of 50 (IRR 3.88, 95% CI 1.81, 8.31), and those over the age of 50 (IRR 2.07, 95% CI 1.06, 4.02). Among individuals that were not diabetic, there was a greater effect of HCV+ on decreased eGFR (IRR 2.72, 95% CI 1.45, 5.10) versus those with diabetes (IRR 1.72, 95% CI 1.00, 2.94). The relationship between HCV+ and eGFR was not meaningfully different across strata of hypertension and Caribbean origin. In formal tests of interaction, sex was the only factor by which the effect of HCV+ varied significantly in fully adjusted analyses (*P* for interaction = .01).

## 4 Discussion

In this multi-center study of Hispanic/Latino individuals, HCV+ was not associated with significantly increased incidence of CKD when defined by eGFR <60 mL/min/1.73 m^2^ or albuminuria. However, when examining changes in eGFR as a separate, pre-specified outcome, HCV+ was significantly associated with decreased eGFR, even after controlling for known demographic, lifestyle, and clinical risk factors. Of note, most changes in eGFR were decreases from ≥90 mL/min/1.73 m^2^ to 60 to 89 mL/min/1.73 m^2^, which is still within a normal range of kidney function.

The most recent KDIGO clinical practice guideline for the prevention, diagnosis, evaluation, and treatment of HCV infection in CKD states that HCV+ individuals are at an increased risk of CKD and ESKD, based on the results of previous analyses.^[5,7,27]^ In comparison, our findings do not support a robust relationship between HCV infection and incident CKD. The results of our analysis may be different due to a number of reasons. First, prior publications varied in their definitions of HCV exposure and cases of CKD, and many studies that have observed strong associations between HCV+ and CKD have defined CKD by ICD codes which may be subject to misclassification, or eGFR thresholds, without regard to albuminuria.^[5,7,28–30]^ As highlighted by Lucas, the heterogeneity in the methods by which both HCV+ and CKD have been defined across many prior investigations of this relationship is likely a key explanation of why previous findings have been inconsistent.^[8]^ This may also explain the contrast between our findings and those of prior investigations.

Additionally, our findings may differ because our analysis focused on a relatively young and healthy cohort of Hispanics/Latinos, with the average age of participants being 47 years. Age is a strong risk factor for declining kidney function and CKD,^[31,32]^ and the age range of this cohort may partially explain why relatively few outcomes were observed; only 6.8% of participants experienced incident CKD at visit 2. Prior works have also suggested that age may modify the relationship between HCV and markers of CKD.^[33,34]^ For example, in a cross-sectional analysis of 15,029 participants of the National Health and Nutrition Examination Survey, Tsui et al found that HCV seropositivity was only significantly associated with albuminuria in higher age groups.^[33]^ Therefore, it is also plausible that we did not observe a robust association between HCV and CKD because our cohort may not have captured the age range in which HCV has a strong effect on kidney function.

We also observed that the effect of HCV+ on longitudinal changes in KDIGO stage-based eGFR varied by sex, with HCV+ conferring a greater risk of worsened eGFR among women. In general, HCV prevalence and associated liver damage is greater among men than women^[35]^ and men with CKD are also more likely to progress to ESKD.^[36]^ However, there has been limited research on sex differences in the relationship between HCV+ and incident CKD.^[34]^ Therefore, prospective investigations of effect measure modification by sex are warranted.

The present study has numerous strengths. HCV+ status was confirmed with measured viral load and thus, those classified as HCV+ were more likely to be representative of chronically infected individuals than participants of previous studies which have classified HCV+ based only on antibody testing. Our definition of incident CKD, with the inclusion of potential albuminuria, is more closely aligned with current guidelines for the diagnosis of CKD. Importantly, this analysis was performed in a large, population-based cohort of Hispanics/Latinos, a growing segment of the American population that has not been well-studied in the context of kidney disease and other conditions.

This study also had notable limitations. The outcome of incident CKD and the component outcomes of decreased eGFR and increased ACR were relatively rare, likely contributing to substantial imprecision in some of our estimates. Furthermore, our analysis may have been subject to selection bias, as it only included participants that were present for visit 2 of the HCHS/SOL study, although previous work has suggested that those lost to follow-up were more likely to be male, US-born, and without a high school diploma than those who remained.^[19]^ A further limitation is that HCHS/SOL is only representative of the communities from which participants were sampled. Although the four HCHS/SOL field centers recruited from states with some of the largest populations of Hispanics/Latinos in the country,^[37]^ it remains a possibility that our results may not be generalizable to all US Hispanics/Latinos. Lastly, this study was unable to stratify HCV+ cases by subtype. Future work could further examine if there are differences in CKD risk associated with specific HCV genotypes and strains.

In summary, in our study of Hispanics/Latinos, HCV+ was associated with declines in eGFR but not incident CKD or increased albuminuria. The results of this study suggest that HCV+ may not be a major risk factor for CKD in this population and priority in the primary prevention of CKD among Hispanics/Latinos may be focused on traditional risk factors. However, treatment with direct-acting antivirals is recommended for all HCV+ including those with established CKD or ESKD.

## Acknowledgments

The authors thank the staff and participants of HCHS/SOL for their important contributions. The Hispanic Community Health Study/Study of Latinos is a collaborative study supported by contracts from the National Heart, Lung, and Blood Institute (NHLBI) to the University of North Carolina (HHSN268201300001I/N01-HC-65233), University of Miami (HHSN268201300004I/N01-HC-65234), Albert Einstein College of Medicine (HHSN268201300002I/N01-HC-65235), University of Illinois at Chicago—HHSN268201300003I/N01-HC-65236 Northwestern Univ), and San Diego State University (HHSN268201300005I/N01-HC-65237). The following Institutes/Centers/Offices have contributed to the HCHS/SOL through a transfer of funds to the NHLBI: National Institute on Minority Health and Health Disparities, National Institute on Deafness and Other Communication Disorders, National Institute of Dental and Craniofacial Research, National Institute of Diabetes and Digestive and Kidney Diseases, National Institute of Neurological Disorders and Stroke, NIH Institution-Office of Dietary Supplements. Dr James Lash is supported by midcareer investigator award in patient-oriented research from the National Institute of Diabetes and Digestive and Kidney Diseases (K24-DK092290).

## Author contributions

**Conceptualization:** Eugenia Wong, Ana C. Ricardo, Nora Franceschini.

**Data curation:** Eugenia Wong, Nora Franceschini.

**Formal analysis:** Eugenia Wong.

**Investigation:** Eugenia Wong.

**Methodology:** Eugenia Wong, Sylvia E. Rosas, Nora Franceschini.

**Supervision:** Nora Franceschini.

**Writing – original draft:** Eugenia Wong.

**Writing – review & editing:** Eugenia Wong, Ana C. Ricardo, Sylvia E. Rosas, James P. Lash, Nora Franceschini.

## Supplementary Material

Supplemental Digital Content
