# Supplementary material for: Hepatitis C infection and chronic kidney disease among Hispanics/Latinos
Source: Medicine (Baltimore). 2021 Dec 10;100(49):e28089. doi: 10.1097/MD.0000000000028089 (PMC8663903; doi:10.1097/MD.0000000000028089)
Supplement: Supplemental Digital Content [file medi-100-e28089-s001.docx]

**Supplemental Digital Content**

Supplement to: Hepatitis C Infection and Chronic Kidney Disease among Hispanics/Latinos

**eTable 1. Changes in eGFR by Stage**

**eTable 2. Effects of Hepatitis C on Incidence of Impaired Kidney Function by eGFRcys**

**eTable 3. Changes in Albuminuria by Stage**

**eTable 1. Changes in eGFR by Stage**

| **Visit 1 eGFR Stage** (N, Row %) | **Visit 2 eGFR Stage** | | | | | |
| --- | --- | --- | --- | --- | --- | --- |
|  | **G1** | **G2** | **G3a** | **G3b** | **G4a** | **Total** |
| **G1** | 6,119 (88.6) | 732 (10.6) | 35 (0.5) | 15 (0.2) | 9 (0.1) | **6,910** |
| **G2** | 1,076 (33.7) | 1,926 (60.3) | 146 (4.6) | 30 (0.9) | 17 (0.5) | **3,195** |
| **G3a** | 18 (7.3) | 112 (45.2) | 71 (28.6) | 39 (15.7) | 8 (3.2) | **248** |
| **G3b** | 6 (10.7) | 2 (3.6) | 11 (19.6) | 24 (42.9) | 13 (23.2) | **56** |
| **G4+** | 2 (9.5) | 1 (4.8) | 3 (14.3) | 2 (9.5) | 13 (61.9) | **21** |
| **Total** | **7,221** | **2,773** | **266** | **110** | **60** | **10,430** |

Cells shaded in red represents participants with worsened eGFR stage at visit 2.

**eTable 2. Effects of Hepatitis C on Incidence of Impaired Kidney Function by eGFRcys**

|  | **Events** | | **Incidence Rate Ratios** | | |
| --- | --- | --- | --- | --- | --- |
|  | **HCV-** | **HCV+** | **Model 1** | **Model 2** | **Model 3** |
| **CKD*** | 882 | 18 | 2.23 [1.22, 4.11] | 1.52 [0.80, 2.91] | 1.58 [0.81, 3.11] |
| **+eGFR Stage**** | 2,276 | 48 | 2.46 [1.67, 3.63] | 1.58 [1.10, 2.26] | 1.50 [1.06, 2.12] |

*Analysis among 8,804 participants without prevalent CKD at visit 1
**Analysis among full cohort of 10,430 participants.

**Model 1:** Crude

**Model 2**: + Demographics (age, center, Caribbean ethnicity, gender, education) **Model 3**: + Comorbidities (diabetes, smoking status, alcohol use, BMI, hypertension, eGFR)

**eTable 3. Changes in Albuminuria by Stage**

| **Visit 1 ACR Stage** (N, Row %) | **Visit 2 ACR Stage** | | | **Total** |
| --- | --- | --- | --- | --- |
|  | **A1** | **A2** | **A3** |  |
| **A1** | 8,809 (94.2) | 510 (5.5) | 34 (0.4) | 9,353 |
| **A2** | 421 (45.8) | 384 (41.7) | 115 (12.5) | 920 |
| **A3** | 27 (17.2) | 43 (27.4) | 87 (55.4) | 157 |
| **Total** | **9,257** | **937** | **236** | **10,430** |

Cells shaded in red represents participants with worsened ACR stage at visit 2.
